# Using a BonE BiOPsy (BeBoP) to determine the causative agent in persons with diabetes and foot osteomyelitis: study protocol for a multicentre, randomised controlled trial

**DOI:** 10.1186/s13063-021-05472-6

**Published:** 2021-08-03

**Authors:** Meryl Cinzía Tila Tamara Gramberg, Rimke Sabine Lagrand, Louise Willy Elizabeth Sabelis, Martin den Heijer, Vincent de Groot, Max Nieuwdorp, Willemijn Kortmann, Elske Sieswerda, Edgar Josephus Gerardus Peters

**Affiliations:** 1 Department of Internal Medicine, Amsterdam Movement Sciences, Amsterdam UMC, Vrije Universiteit Amsterdam, De Boelelaan 1117, Amsterdam, The Netherlands; 2 Department of Rehabilitation Medicine, Amsterdam Movement Sciences, Amsterdam UMC, Vrije Universiteit Amsterdam, De Boelelaan 1117, Amsterdam, The Netherlands; 3 Department of Internal Medicine, Amsterdam Movement Sciences, Amsterdam UMC, Vrije Universiteit Amsterdam, Academisch Medisch Centrum, De Boelelaan 1117, Amsterdam, The Netherlands; 4 Department of Internal Medicine, Noordwest Ziekenhuisgroep, Wilhelminalaan 12, Alkmaar, The Netherlands; 5 Department of Medical Microbiology, Medical Cemtre Utrecht, University of Utrecht, Heidelberglaan 100, Utrecht, The Netherlands

**Keywords:** Osteomyelitis in diabetic foot, Bone biopsy, Antibacterial therapy

## Abstract

**Background:**

Diabetic foot osteomyelitis (DFO) poses a major disease burden. It can generally be treated with long-term antibacterial therapy. International guidelines recommend to base antibacterial therapy choices on percutaneous bone biopsy culture, while in practice, therapy is frequently based on (less invasive) ulcer bed cultures. It is currently unknown if treatment outcomes of DFO differ depending on the chosen diagnostic strategy.

**Methods:**

The BeBoP trial is a multicentre; randomised controlled; physician-, researcher- and subject-blinded; clinical trial comparing two diagnostic strategies in persons with DFO. Culture-directed antibacterial therapy will be based on either percutaneous bone biopsy culture results (intervention group) or ulcer bed biopsy culture results (comparison group). We will enrol 80 subjects with diabetes mellitus (≥ 18 years) and DFO, and we will use block randomisation stratified per centre to randomise them in a 1:1 allocation. The primary outcome is remission of DFO 12 months after enrolment. The secondary outcomes are the time to remission, signs of inflammation or ulceration at the primary location of infection at 6 and 12 months, microbiological and molecular profiles of culture outcomes, surgical interventions including amputation, total antibacterial therapy duration, infection-free survival days, adverse events, quality of life and survival. We will compare the outcomes by intention-to-treat and per-protocol analysis.

**Discussion:**

We aim to compare clinical remission in persons with DFO treated with antibacterial therapy based on either percutaneous bone biopsy culture results or ulcer bed biopsy culture results.

**Trial registration:**

Netherlands Trial Register NL 7582. Registered on 05 March 2019

**Supplementary Information:**

The online version contains supplementary material available at 10.1186/s13063-021-05472-6.

## Background

Diabetic foot osteomyelitis (DFO) poses a major social, psychological and economic disease burden in persons with diabetes mellitus (DM). Roughly 34% of persons with DM will develop a foot ulcer at some point in their life [1]. Foot ulcers are complicated by a foot infection in up to 60% of cases and approximately 20% of persons with DM and a foot infection have osteomyelitis [2–4]. Osteomyelitis is a leading cause of surgical lower extremity amputation in over 80% of persons with DM with subsequent loss of quality of life [5, 6].

Defining appropriate antimicrobial therapy directed to the causative bacteria in DFO is challenging. A surgically or percutaneously acquired bone sample for culture is the reference standard to diagnose DFO and to determine the causative bacteria and their susceptibility. A percutaneous bone biopsy should be acquired through aseptic skin adjacent to the ulcer, not through the ulcer bed [7, 8]. Some clinicians find this method cumbersome or too invasive and base their choice of antibacterial therapy on cultures from a superficial ulcer swab or ulcer bed biopsy. Obtaining material from the ulcer frequently results in culturing non-related bacteria that easily colonise ulcers rather than causative pathogens of DFO [9]. Treatment based on cultures from material taken from or through the ulcer may therefore lead to suboptimal antibacterial therapy. In a prospective cohort study, in 13% of subjects, antibacterial therapy did not cover all causative bacteria, and 18.5% of subjects were treated with antibacterial therapy with an overly broad spectrum of activity when treatment was based on a biopsy through the ulcer bed instead of a percutaneous bone biopsy [10]. A retrospective cohort study showed that subjects treated with antibacterial therapy targeted at bacteria of bone biopsy cultures heal in 80%, compared with 50% of subjects treated with antibacterial therapy targeted at bacteria of superficial ulcer swab cultures [11]. No prospective trial data are available to compare the true difference in clinical outcomes between the two diagnostic strategies.

Another challenge in adequately treating DFO is the lack of knowledge about the type and diversity of bacteria in infected bone. It remains unclear, for instance, if anaerobic and skin flora can act as pathogens in DFO [12].

## Methods

### Aim

#### Primary objective

1. To prospectively compare remission of osteomyelitis in persons with DFO treated based on bone biopsy cultures (intervention group) versus those treated based on ulcer bed cultures (comparison group).

#### Secondary objectives

2.1 To compare time to remission, signs of inflammation at the primary location of infection at 6 and 12 months, surgical interventions including amputation, total antibacterial therapy duration, infection-free survival days, adverse events, quality of life and survival in persons with DFO treated with antibacterial therapy targeted at bone biopsy cultures versus antibacterial therapy targeted at ulcer bed biopsy cultures.

2.2 To compare culture results of the diagnostic strategies and to explore if certain microbiota profiles are related to the primary outcome.

### Design

This study is a multicentre; randomised; physician-, researcher- and subject-blinded; clinical trial which will be undertaken in hospitals in The Netherlands. In this RCT, we will investigate if bone biopsy (the intervention) is superior compared to ulcer bed biopsy (the control) for culture based treatment. The trial is registered in the Dutch trial registry: Trial NL7582. We used the SPIRIT reporting guidelines to draft this study protocol [13]. Table 1 shows the study schedule.

**Table 1:** SPIRIT figure on enrolment, interventions and assessments

	Study period					
	**Enrolment**	**Allocation**	**Post-allocation**			**Close-out**
Time points*	***−T***_**1**_	***T***_**0**_	***T***_**1**_	***T***_**2**_	***T***_**3**_	***T***_***x***_
**Enrolment:**						
**Eligibility screen**	X					
**Informed consent**	X					
**Allocation**		X				
**Intervention:**						
***Bone biopsy and ulcer bed biopsy***		X				
**Assessments:**						
***Baseline variables:***						
***Demographic data***		X				
***Plain X-ray and/or MRI and/or FDG-PET***		X				
***Foot temperatures***		X	X	X	X	
***Peripheral arterial status***		X	X	X	X	
***Standard laboratory tests***		X	X	X	X	
***Microbiology bone/ulcer bed biopsy***		X				
***Outcome variables:***						
***Remission of osteomyelitis***					X	
***Time to remission***			X	X	X	
***Signs of inflammation***		X	X	X	X	
***Ulceration at the primary location of infection***			X	X	X	
***Microbiological and molecular profiles of bone and ulcer bed cultures***		X				
***Occurrence of any surgical amputation***			X	X	X	
***Total antibacterial therapy duration***			X	X	X	
***Infection-free survival days***			X	X	X	
***Quality of life***		X		X	X	
***Survival***			X	X	X	
***Adverse events***			X	X	X	

*Specification of time points:

*−T*_1_ before enrolment; *T*_0_ day 1; *T*_1_ after empirical and guided therapy (± 7 weeks after enrolment); *T*_2_ 6 months after enrolmen; *T*_3_ 12 months after enrolment; *T*_*x*_ ≥ 12 months

### Setting

We will include 80 subjects (40 subjects in the control and intervention groups) that present as either outpatient or inpatient at tertiary care centre Amsterdam University Medical Centre location VUmc or AMC or secondary care centre Noordwest Hospital group location Alkmaar or Alrijne Hospital Group Leiderdorp. All eligible patients will be informed and asked to participate and for additional consent for ancillary studies by their treating physician. The informed consent procedure and enrolment including randomisation will be performed by a member of the study team. Semi-annual inclusion targets are formed. If inclusion targets are not reached, we will extend the number of participating centres.

### Participants

Eligibility criteria are shown in Table 2.

**Table 2:** Trial registration data set

Data category	Information
Primary registry and trial identifying number	Netherlands Trial Register NL 7582
Date of registration in primary registry	2019-03-05
Secondary identifying numbers	Dutch Diabetes Research Foundation (*Diabetes Fonds*) number 2017.82.014
Source(s) of monetary or material support	Dutch Diabetes Research Foundation
Primary sponsor	Amsterdam University Medical Centers, location VUmc, Amsterdam, The Netherlands Address: De Boelelaan 1117, 1081 HV, Amsterdam +31 20 4444444
Secondary sponsor(s)	
Contact for public queries	Meryl Gramberg, m.gramberg@amsterdamumc.nl Edgar Peters, e.peters@amsterdamumc.nl
Contact for scientific queries	Meryl Gramberg, m.gramberg@amsterdamumc.nl Edgar Peters, e.peters@amsterdamumc.nl
Public title	Using a BonE BiOPsy to Determine the Causative Agent in Persons with Diabetes and Foot Osteomyelitis (BeBoP).
Scientific title	Using a BonE BiOPsy to Determine the Causative Agent in Persons with Diabetes and Foot Osteomyelitis (BeBoP): Study Protocol for a Multicentre, Randomised Controlled Trial.
Countries of recruitment	The Netherlands
Health condition(s) or problem(s) studied	Foot osteomyelitis in persons with diabetes, bone biopsy, ulcer bed biopsy
Intervention(s)	Culture-directed antibacterial therapy based on percutaneous bone biopsy culture results (intervention group)
	Culture-directed antibacterial therapy based on ulcer bed biopsy culture results (comparison group)
Key inclusion and exclusion criteria	Sexes eligible for study: all; Accepts healthy volunteers: no
	Eligibility criteria are: • ≥ 18 years • Being able to give informed consent and willing to comply with the research protocol • Type 1 or type 2 DM • Moderate or severe foot infection extending to the anatomical plane of fascia, muscle, tendon or bone (International Working Group on the Diabetic Foot (IWGDF) infection grade 3 or 4 with osteomyelitis) [8]. • Subjects will have at least one of the following signs of osteomyelitis: a positive probe to bone test [8], abnormalities on plain X-ray suggestive for osteomyelitis [14], erythrocyte sedimentation rate (ESR) ≥70 mm/h (without another explanation for the elevated ESR), signs of osteomyelitis on MRI and/or FDG-PET/CT- scan [14], or positive microbiological, molecular culture results, or histology of a recent percutaneous bone biopsy performed before inclusion.
	Exclusion criteria: 1. Likely to undergo complete surgical bone debridement or amputation. Subjects that (are likely to) undergo surgery within 72 h after enrolment for other reasons, e.g. abscesses, compartment syndrome, partial bone resection, are not excluded. 2. Presence of critical limb ischemia which cannot be corrected. Subjects that (are likely to) undergo surgical or percutaneous revascularisation are not excluded. 3. Severely immunocompromised (as judged by the treating physician, e.g. neutropenia due to chemotherapy, HIV infection with CD4-count of < 200/μl) 4. Pregnant or lactating 5. Unable to give informed consent 6. Unlikely to live at least 1 year 7. Unable or unwilling to follow protocol requirements (with exception of rectal swabs) 8. Received systemic antimicrobial therapy within 1 week before enrolment
Study type	Interventional
	Allocation: randomised intervention model. Physician, researcher and subjects are blinded for the culture results of the not-assigned treatment strategy.
	Primary purpose: diagnostic trial
Date of first enrolment	February 2019
Target sample size	80
Recruitment status	Recruiting
Primary outcome(s)	Remission of osteomyelitis at 12 months, whereby remission of osteomyelitis is defined as: an epithelialised ulcer for 28 consecutive days and absence of local inflammation and/ or stabilisation or improvement of radiologic abnormalities.
Key secondary outcomes	Time to remission, signs of inflammation or ulceration at the primary location of infection at 6 and 12 months, microbiological and molecular profiles of culture outcomes, occurrence of any surgical amputation of the primary affected leg, total antibacterial DFO therapy duration, infection-free survival days, quality of life, survival and adverse events at 12 months.

Subjects that (are likely to) undergo surgery within 72 h after enrolment for other reasons (e.g. abscesses, compartment syndrome, partial bone resection) and subjects that (are likely to) undergo surgical or percutaneous revascularisation are not excluded.

### Randomisation

**Fig. 1:**
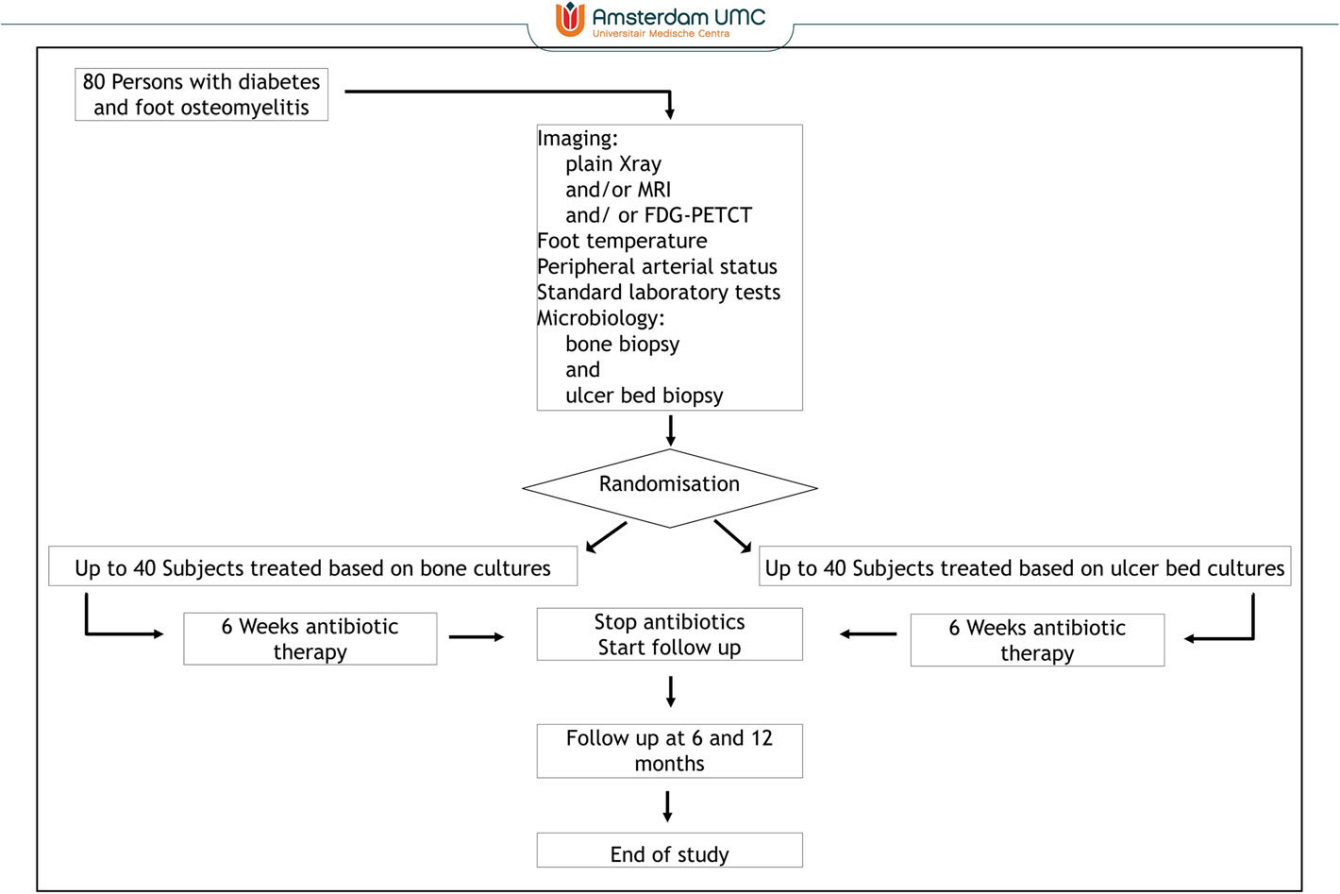
Flow chart of study design

Randomisation will take place after subjects give informed consent. We will allocate subjects to each group (intervention or comparison diagnostic strategy) by a computerised randomisation programme (Castor) that generates a block randomisation (block size four) with a random (1:1) allocation sequence and stratified per centre (see Fig. 1).

### Study procedures

Percutaneous collection of a specimen of bone and tissue biopsy of the ulcer bed takes place in all included subjects before initiation of antibacterial therapy. Only the culture results of the randomised diagnostic strategy will be displayed in the subjects’ medical record.

#### Percutaneous bone biopsy

Infected bone will be identified using plain X-ray, MRI or FDG-PET/CT scan based on previously published studies [14, 15]. Under aseptic conditions, we will obtain a piece of infected bone with a 11-gauge needle through anesthetised skin adjacent to the ulcer. We will divide the bone sample into two: one for culture and one for molecular microbiology.

#### Ulcer bed culture

We will obtain an ulcer bed tissue biopsy for culture in each subject. After rinsing the ulcer with sodium chloride 0.9%, a small specimen of soft tissue will be taken from the ulcer bed.

#### Handling of specimens

Specimens will be transported to the microbiology laboratory. The specimens will be examined by standard culture techniques for identification and antimicrobial sensitivity. Standard culture techniques include inoculating the sample on agar plates and in broth, followed by incubation in aerobic and anaerobic environment. Bacteria will be identified and their susceptibility will be defined both according to local laboratory protocols.

#### Antibacterial therapy

After we obtain the biopsies, we will start predefined empiric antibacterial therapy (Table 3). When bone biopsy or ulcer bed cultures become available, the treating physician switches to targeted therapy depending on bacterial susceptibility and subject characteristics and prescribes this therapy during the visit to the outpatient clinic and gives instructions to the patient. (Table 3). To reduce variation in antibacterial therapies, the treating physician will discuss any non-preferred regimen with the study team. The targeted antibacterial therapy will be aimed at either the bone biopsy or ulcer culture result depending on the treatment strategy allocation of the subject (see blinding and unblinding below). All bacteria cultured will be considered pathogenic and will be treated with targeted therapy. All subjects will be treated with targeted antibacterial therapy for 6 weeks (±5 days).

**Table 3:** Preferred antibiotic regimen

Organism	Intravenous antibiotic	Oral antibiotic
Empiric antibiotic regimen In case of beta lactam allergies:	Amoxicillin/clavulanic acid 1200 mg QID Ceftriaxone 2 g qd + metronidazole 500 mg TID Clindamycin 600 mg TID + ciprofloxacin 400 mg TID	Amoxicillin/clavulanic acid 625 mg TID Clindamycin 600 mg TID AND Ciprofloxacin 750 mg BID
*Staphylococcus aureus* (MSSA) and methicillin-sensitive coagulase-negative staphylococci	Flucloxacillin 6 g/24 h	Clindamycin 600 mg TID Trimethoprim/sulfamethoxazole (co-trimoxazole) 960 mg BID
*Staphylococcus aureus* (MRSA) and methicillin-resistant coagulase-negative staphylococci	Vancomycin (follow local antibiotic guideline for dosage) Teicoplanin (follow local antibiotic guideline for dosage)	Clindamycin 600 mg TID Trimethoprim/sulfamethoxazole (Co-trimoxazole) 960 mg BID Linezolid 600 mg BID Moxifloxacin 400 qd
Beta haemolytic streptococci	Penicillin 12 mil U/24 h	Clindamycin 600 mg TID Doxycycline 200 mg BID
Enterococci	Amoxicillin 6 g/24 h Vancomycin (follow local antibiotic guideline for dosage)	Amoxicillin 1000 mg TID Linezolid 600 mg BID
Enterobacteriaceae	Amoxicillin 6 g/24 h Ceftriaxone 2 g qd Ciprofloxacin 400 mg TID	Ciprofloxacin 750 mg BID Trimethoprim/sulfamethoxazole (co-trimoxazole) 960 mg BID
Non-fermenting gram-negative rods, e.g. *Pseudomonas*, *Enterobacter*	Ceftazidime 3 g/24 h Ciprofloxacin 400 mg TID	Ciprofloxacin 750 mg BID
Anaerobes (gram-negative, e.g. *Bacteroides*)	Metronidazole 500 mg TID Clindamycin 600 mg TID	Metronidazole 500 mg TID Clindamycin 600 mg TID
Anaerobes (gram-positive, e.g. *Cutibacterium acnes*, *Finegoldia*)	Penicillin 12 mil U/24 h Doxycycline 200 mg BID	Amoxicillin 1000 mg TID Doxycycline 200 mg BID

The dosages and intervals are not adjusted to renal function. *MSSA* methicillin-sensitive *S. aureus*, *MRSA* methicillin-resistant *S. aureus*, *BID* two times per day, *TID* three times per day, *qd* daily, *QID* four times per day

#### Questionnaires

At the start, after 6 and 12 months, we will ask subjects to fill out questionnaires regarding their quality of life, physical capacity and disease burden.

### Standard care

We offer the following standard care to all subjects starting at their first visit. We will provide adequate biomechanical pressure offloading of the ulcer site, assessed by in-shoe pressure measurement. We will screen for the presence of peripheral artery disease by palpation of peripheral arterial pulsations. If any pedal pulsation is absent, we will perform ankle-brachial index and toe pressure measurements. If these are abnormal, we will perform a CT angiography and, if required, offer the subject surgical or percutaneous revascularisation. At each follow-up visit, we will measure foot skin temperatures, take ulcer measures and if required ulcer debridement will take place and ulcer dressings will be prescribed. If necessary other concomitant treatments will be permitted. Standard care will take place during regular weekly or two-weekly visits to the diabetic foot and will continue if required after cessation of antibacterial therapy. During these visits, we will monitor subjects adherence to treatments and protocol and we will customise therapy (if necessary and possible), so adherence is as easy as possible.

### Follow-up

Follow-up and assessment of outcome measures will take place at the end of the 6 weeks of antibacterial therapy (± 3 days) and at 6 (± 2 weeks) and 12 months (± 2 weeks) after enrolment. During follow-up visits, we will register adverse events and we will report adverse events bundled or immediately (in case of serious events) to the sponsor.

### Blinding and unblinding

The physician, researcher and subject will be blinded to the culture results of the diagnostic strategy the subject is not allocated to. Only randomised cultures will become available in the patients’ medical record. The medical microbiologist is not blinded for any of the culture results of the diagnostic strategies. Both cultures will be displayed in a separate computer programme (GLIMS) only accessible by microbiologists. If the treating physician observes that the infection deteriorates (e.g. because of an increase of ulcer size, foot temperatures, pain, systemic inflammation markers and fever) in the first 7 days of guided therapy, or if the bacterial culture of the allocated diagnostic strategy is sterile, the treating physician asks the clinical microbiologist to unblind the previously blinded non-allocated diagnostic strategy. Antibacterial therapy will be adjusted based on the now unblinded diagnostic strategy. If highly resistant micro-organisms are cultured in the blinded diagnostic strategy that require infection control measures during subject visits, the clinical microbiologist will give advice on such measures to the treating physician without disclosing details of the blinded culture results (e.g. methicillin resistant *Staphylococcus aureus*). Subjects with sterile microbiology in the cultures of both of the diagnostic strategies will be excluded from the study, as these subjects are considered not to have osteomyelitis.

After closing, the trial cultures of the blinded non-allocated diagnostic strategy will become available for the researchers. Cultures of both diagnostic strategies can then be compared and analysed.

### Outcomes and definitions

The primary outcome measure will be remission of osteomyelitis at 12 months, whereby remission of osteomyelitis is defined as an epithelialised ulcer for 28 consecutive days and absence of local inflammation and/ or stabilisation or improvement of radiologic abnormalities. Secondary outcome measures at 6 and 12 months will be time to remission (in days measured during follow-up), signs of inflammation (redness, warmth, swelling, pain and purulent exudate measured as being present or not during follow-up), ulceration at the primary location of infection (measured as being present or not), occurrence of any surgical amputation of the primary affected leg during the study period, total antibacterial therapy duration (mean in days), infection-free survival days (median measured in days during follow-up), quality of life (median score of questionnaire), survival (during follow-up) and the occurrence of adverse events during the study (qualitative). For quality of life, we will use the following validated questionnaires in Dutch: Problem Areas in Diabetes (PAID-NL) [16], Short Form-36 Health Survey (SF-36) [17], and Utrecht Scale for Evaluation of Rehabilitation-Participation (USER-P) [18]. We will register any serious adverse events ((S)AEs), i.e., AEs grade 3 or higher according to Common Terminology Criteria for Adverse Events (CTCAE) v5.0 [19]. We will also record any AE related to the intervention or if such AE led to dose adjustment or cessation of antibacterial therapy. We will evaluate if an event is related, possibly related or not related to the trial intervention. Trial related events are side effects of antibacterial therapy up to 12 weeks after cessation of therapy and the occurrence of infection or bleeding within 24 h after ulcer bed or bone biopsy. Other events will be evaluated independently and classified accordingly.

The diagnostic strategy will be considered a failure if (1) a patient is not in remission after 12 months, (2) unblinding because of sterile cultures of the randomised strategy, (3) unblinding is required in the first 7 days after initiation of guided therapy because of deteriorating infection and (4) the treating physician decides that prolongation of antibacterial therapy is needed longer than the predefined 6 weeks of treatment.

**Fig. 2:**
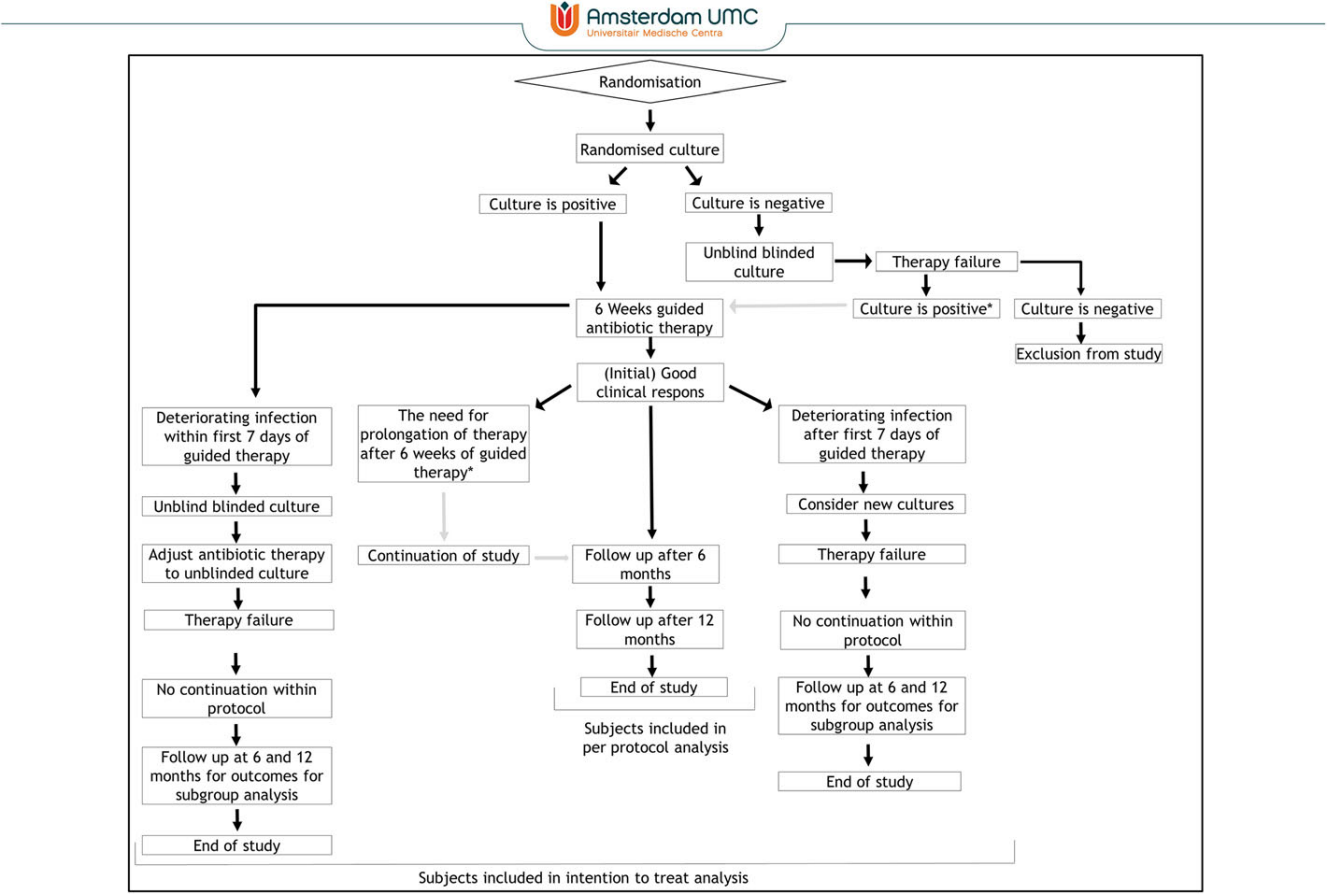
Flow chart of study courses. *We will perform additional analysis allowing for antibacterial therapy longer than 6 weeks and treatment based on unblinded cultures

We will perform two additional per-protocol analyses: (1) allowing for treatment based on unblinded cultures and (2) treatment based on randomised cultures, but the need for continuation longer than 6 weeks. In the first situation, treatment based on one of the strategies might be safe in case of sterile cultures of the other strategy and in the second a longer treatment might suffice to achieve remission. These are possible outcomes we want to anticipate (Fig. 2).

### Data collection

The treating physician or a trained member of the research team will collect data at each subject visit at the diabetic foot clinic. At inclusion, we will give subjects a study number to pseudo-anonymise data. Source data will be recorded in the electronic patient file. Study data will be entered in an electronic data capture system (Castor EDC) [20] and screen data of subjects screened for eligibility, including reasons for exclusion in a different database. Data will be pseudo-anonymised. After the trial, data will be maintained and will only be accessible for the study team.

### Sample size

The number of subjects is chosen based on the following assumptions: the trial has a dichotomous primary outcome. We assume a remission rate of 50% in subjects treated with antibacterial therapy targeted to ulcer cultures versus 80% in the group treated with antibacterial therapy targeted to bone cultures (remission rates based on Senneville, 2008) [11], a power of 0.8 and an alpha of 0.05, and an enrolment ratio of 1, the calculated sample size is 76 for a superiority assumption. A sample size of 80 includes an anticipated dropout for unknown reasons of 4 subjects.

### Analysis

We will describe demographic and baseline variables and display mean, median, standard error, 95% confidence interval, or frequencies when appropriate. For our primary analysis, we will evaluate the effect of both diagnostic strategies with an intention-to-treat and per-protocol analysis (objective 1). We will present relative risks for the primary outcome. To further analyse remission in the intervention and control group, we will use logistic regression (objective 1). To analyse the data for the dichotomous variables (signs of inflammation at the primary location of infection at 6 and 12 months, surgical interventions including amputation and adverse events), we will also use logistic regression (objective 2.1). We will use regression analyses for the continuous outcome variables of the total antibacterial therapy duration and infection-free survival days (objective 2.1). We will correct the abovementioned analyses for possible covariates (e.g. medical centres). We will present Kaplan-Meier curves for time remission and survival and will analyse these data with Cox regression (objective 2.1). Quality of life will be measured at three different moments in time for each subject. Since these measurements are not independent, we will compare differences in change of quality of life with linear mixed models. We will also display relative and absolute risks for adverse events related to the intervention or antibacterial therapy and for survival (objective 2.1).

We will perform intention-to-treat analyses of our primary and secondary outcomes of all subjects in our study. We will perform per-protocol analyses on all subjects who followed protocol comparing final treatment based on bone or ulcer bed biopsy cultures. We will then compare outcomes of our intention-to-treat analyses and per-protocol analyses. We will interpret the results taking into account the additional analyses regarding the amount of subjects in whom antibacterial therapy was altered because of sterile culture results in the randomised diagnostic strategy and patients in which duration of treatment was prolonged beyond 6 weeks of therapy.

We will perform separate sensitivity analyses on subgroups of subjects in whom antibacterial therapy was prolonged, who had a deteriorating infection before and after 7 days of guided treatment. We will also perform sensitivity analysis on subjects with a negative bone biopsy.

We will describe microbiological profiles of culture outcomes and group bacteria according to type or clinical hallmark during infection. We will dichotomise differences between microbiological cultures and we will analyse this dichotomised variable with logistic regression (objective 2.2).

We will build a logistic prediction model with backward selection to assess which microbiota profiles are predictive of our primary outcome (objective 2.2).

We plan to publish the results of this trial in a peer-reviewed journal.

### Patient and public involvement

The Dutch Diabetes Research Foundation will distribute our findings to patient associations. We will gather spontaneous suggestions from patients and patient associations in response to our findings.

## Discussion

The BeBoP trial addresses some of the knowledge gaps concerning DFO. The most important one is which diagnostic strategy to determine the causative agent in persons with DFO leads to the highest remission rate. This study will also provide insight in other differences between treatments based on either bone or ulcer bed biopsy cultures, e.g. time to remission, surgical interventions and quality of life. Outcomes will help to optimise patient care and to limit resources needed for diagnosis and therapy.

We choose this study set-up to mimic everyday clinical practice where usually one of the diagnostic strategies is deployed to diagnose DFO. The prospective nature of the study avoids the inherent bias of previous retrospective studies on this subject.

The physician, researcher and subject will be blinded for one of the culture results. By blinding the culture outcome the subject did not randomise for, we can avoid that choice of antibacterial therapy is influenced by the not-allocated culture outcomes. We will use antibacterial therapy from a set list to reduce physician preferences in choosing antibacterial therapy, to minimise confounding factors including treatment with unnecessarily broad-spectrum antibacterial medication and to standardise treatment among treating physicians.

We will conduct this study in one high-income country. Extrapolation of results must be done carefully since type of pathogens and resources differ among regions over the world.

As with most of the available diagnostic tests, a bone biopsy is not 100% sensitive and there is a chance of sampling error. This chance is higher in bone biopsy than ulcer bed biopsy. To correct for this possible bias, we perform additional analyses allowing subjects with sterile cultures to be treated based on the diagnostic strategy the subject did not randomise for.

We will perform both diagnostic strategies, so we can compare cultures of both strategies in one person. This can give insight in why outcomes might differ between strategies.

A better determination of the causative agent in DFO will enable a more effective use of resources, and to more adequately targeted antibiotics. Better antimicrobial treatment will likely lower the risk of amputation and infection relapse, thereby increasing quality of life and to lower consumption of (broad-spectrum) antibacterial therapy, leading to lower rates of antibacterial resistance in patients, clinics and the community. Every possible outcome of this trial will have implications for current everyday practice.

## Trial status

Protocol issue date: May 2021, Version 7.

The first participant of the study has been included at the 20th of February 2019. The final inclusion is expected at the beginning of 2023, and in 2024, the trial is due to be completed.

## Supplementary Information

**Additional file 1:.** Appendix 1

**Additional file 2:.** Appendix 2

**Additional file 3:.** Reporting checklist for protocol of a clinical trial.

## Data Availability

We plan to share anonymised study data in a data repository. We will only share data used for published articles.

## Abbreviations

AE: Adverse event
DFO: Diabetic foot osteomyelitis
DM: Diabetes mellitus
ESR: Erythrocyte sedimentation rate
IRB: Institutional Research Board
IWGDF: International Working Group on the Diabetic Foot
PAID-NL: Problem Areas in Diabetes Questionnaire in Dutch
(S)AE: (Serious) adverse event
SF-36: Short Form-36
USER-P: Utrecht Scale for Evaluation of Rehabilitation-Participation
WMO: Medical Research Involving Human Subjects Act (in Dutch: Wet Medisch wetenschappelijk Onderzoek met Mensen)
