# Supplementary material for: Using a BonE BiOPsy (BeBoP) to determine the causative agent in persons with diabetes and foot osteomyelitis: study protocol for a multicentre, randomised controlled trial
Source: Trials. 2021 Aug 3;22:517. doi: 10.1186/s13063-021-05472-6 (PMC8335883; doi:10.1186/s13063-021-05472-6)
Supplement: Supplementary file 1 — Additional file 1:. Appendix 1 [file 13063_2021_5472_MOESM1_ESM.pdf]

APPENDIX 1

Model informed consent form

VUmc, De Boelelaan 1118,
Amsterdam

Afdeling Interne Geneeskunde

M. Gramberg/R. Lagrand
(onderzoekers)

Kamer ZH3A 74

Tel: 0625716415

m.gramberg@amsterdamumc.nl
/r.lagrand@amsterdamumc.nl

Datum:	17-6-2020
Ons kenmerk:	Proefpersoneninformatiebrief V7
Betreft:	Proefpersoneninformatie medisch wetenschappelijk onderzoek: **Wat veroorzaakt botinfecties bij personen met diabetes en hoe lang moet de infectie worden behandeld? BeBoP-studie.**

Geachte heer/mevrouw,

Uw behandelend arts heeft bepaald dat u in aanmerking komt voor deelname aan het onderzoek.

Wij vragen u om mee te doen aan een medisch-wetenschappelijk onderzoek. Meedoen is vrijwillig. Om mee te doen is wel uw schriftelijke toestemming nodig. U ontvangt deze brief omdat u een infectie van het bot heeft en u behandeld zult gaan worden met antibiotica. Voordat u beslist of u wilt meedoen aan dit onderzoek, krijgt u uitleg over wat het onderzoek inhoudt. Lees deze informatie rustig door en vraag de onderzoeker uitleg als u vragen heeft. U kunt ook de onafhankelijk deskundige, die aan het eind van deze brief genoemd wordt, om aanvullende informatie vragen. U kunt er ook over praten met uw partner, vrienden of familie.

Verdere informatie over meedoen aan zo'n onderzoek vindt u op www.rijksoverheid.nl/mensenonderzoek. We willen u vragen uiterlijk binnen 7 dagen te beslissen of u wilt deelnemen aan dit onderzoek.

Met vriendelijke groet,

Edgar Peters, hoofdonderzoeker/internist

Afdeling Interne Geneeskunde.

Amsterdam UMC, locatie VUmc, De Boelelaan 1118, Amsterdam

Bijlagen:

A. Contactgegevens

B. Informatie over de verzekering

C. Schema onderzoekshandelingen / omschrijving onderzoekshandelingen

D Toestemmingsformulier(en)

1. Algemene informatie

Dit onderzoek is opgezet door de diabetische voetwondenpoli in VUmc en wordt gedaan door artsen op diabetische voetenpoli's in diverse ziekenhuizen in Nederland.

Er zullen 80 proefpersonen meedoen.

De medisch-ethische toetsingscommissie in VUmc heeft dit onderzoek goedgekeurd. Algemene informatie over de toetsing van onderzoek vindt u op de website www.rijksoverheid.nl/mensenonderzoek.

2. Doel van het onderzoek

Het doel van dit onderzoek is te bepalen op welke manier de ziekmakende bacterie in het bot kan worden aangetoond. Daarnaast wordt onderzocht hoe lang de behandeling met antibiotica (geneesmiddel tegen de ziekmakende bacterie) moet duren. Ook wordt gekeken wat het effect is van de behandeling op bacteriën in de darm.

3. Achtergrond van het onderzoek

Welke bacterie veroorzaakt de infectie?

Een infectie van het bot bij een voetwond bij diabetes moet behandeld worden om uitbreiding te voorkomen. Die behandeling wordt gedaan met een medicijn dat de ziekmakende bacterie doodt. Zo'n medicijn wordt een antibioticum genoemd. De bacterie die de infectie veroorzaakt, bepaalt welk antibioticum nodig is. Het is belangrijk om te weten welke bacterie

de infectie veroorzaakt. Om te onderzoeken welke bacterie de oorzaak is, moet weefsel van de wond of van het zieke bot worden onderzocht. Dit kan door een stukje weefsel diep uit de wond of een stukje uit het bot te halen. Welke methode het beste werkt, het botbiopt of de weefselkweek, is tot nu toe nog niet goed onderzocht.

Gebruikelijke behandeling van infectie van het bot op dit moment:

Het is nog niet goed onderzocht hoe lang de infectie van het bot actief blijft. De standaardbehandeling duurt nu minimaal 6 weken.

Dit houdt in dat bij start van de behandeling er eerst een aantal onderzoeken worden gedaan, die standaard zijn bij mensen met een botinfectie, namelijk: lichamelijk onderzoek inclusief lichaamstemperatuur, röntgenfoto, MRI-scan (indien er op basis van de andere onderzoeken onduidelijkheid is over de diagnose of locatie van de botinfectie), bloedonderzoek, temperatuurmeting van de huid van de voeten en voetdrukmeting. Er wordt zowel een weefselkweek als een botbiopt gedaan. Gedurende de behandeling wordt u elke 2-3 weken gezien op het diabetische voetenspreekuur van uw ziekenhuis.

Na zes weken beslist de dokter of u nog doorbehandeld moet worden. Als de infectie niet meer actief is wordt er gestopt met behandelen.

Wat is het effect van de behandeling met antibiotica op de bacteriën in het maagdarmstelsel?

Ieder mens heeft bacteriën in zijn darm. Deze bacteriën hebben een rol bij het verteren van voedsel en bij afweer tegen ziekten. Antibiotica hebben invloed op de deze bacteriën. Het is niet bekend wat de invloed van het korter of langer gebruik van bepaalde soorten antibiotica is op de bacteriën in de darm.

4. Wat meedoen inhoudt

Als u meedoet, duurt dat totaal ongeveer 12 maanden voor u.

In deze wetenschappelijke studie worden onderzoeken met elkaar vergeleken die in de dagelijkse praktijk al worden gebruikt. Als u besluit mee te doen dan betekent dat dat u dezelfde behandeling (met antibioticum) krijgt als wanneer u niet mee zou doen.

Bezoeken en metingen anders dan gebruikelijke zorg

Er wordt geloot of u op basis van de weefselkweek of op basis van het botbiopt met antibiotica behandeld wordt. Een deel van de deelnemers zal extra onderzoeken krijgen. Indien u hiervoor kiest, krijgt u aan het begin van de studie een PET/CT scan en MRI. Ook wordt er in dat geval na 3 of 6 weken (afhankelijk van loting) nogmaals een PET/CT scan, MRI en een botbiopt gedaan. Hiermee wordt bepaald of de infectie nog actief is.

U kunt er ook voor kiezen om deze aanvullende onderzoeken aan het begin en na 3 of 6 weken te weigeren. Dit kunt u aangeven op het toestemmingsformulier.

Als u mag stoppen met de behandeling (het antibioticum) zien we u na 6 en 12 maanden nogmaals ter controle van de voeten. Deze controles zullen samenvallen met gebruikelijke controles op de diabetische voetenpoli.

Daarnaast wordt u gevraagd voor start van antibiotica, na staken van antibiotica en bij de controle na 6 en 12 maanden zelf een rectale swab af te nemen (u gaat met een wattenstokje een klein stukje het rectum in en doet het stokje vervolgens in een daarvoor bestemd potje). Als u ervoor kiest om niet deel te nemen aan dit deel van het onderzoek, dan kan u wel gewoon deelnemen aan de rest van het onderzoek. U kunt dit aangeven op het toestemmingsformulier.

Tenslotte wordt u op drie momenten (begin, eind en na 6 maanden) gevraagd een aantal vragenlijsten over uw gezondheid in te vullen.

5. Wat wordt er van u verwacht?

Om het onderzoek goed te laten verlopen is het belangrijk dat u zich aan de volgende afspraken houdt.

De afspraken zijn dat u:

- niet ook nog aan een ander medisch-wetenschappelijk onderzoek meedoet.
- afspraken voor bezoeken nakomt.

Het is belangrijk dat u contact opneemt met de onderzoeker:

- voordat u andere geneesmiddelen gaat gebruiken. Ook als dat homeopathische geneesmiddelen, natuurgeneesmiddelen, vitaminen en/of geneesmiddelen van de drogist zijn.
- als u in een ziekenhuis wordt opgenomen of behandeld.
- als u plotseling gezondheidsklachten krijgt.
- als u niet meer wilt meedoen aan het onderzoek.
- als uw contactgegevens wijzigen.

Zwangerschap

Vrouwen die zwanger zijn of borstvoeding geven, kunnen niet meedoen aan dit onderzoek. Ook mogen vrouwen niet zwanger worden tijdens het onderzoek.

Zowel het gebruik van antibioticum als de PET/CT-scan kan namelijk gevolgen hebben voor een ongeboren kind. Het is belangrijk dat u dit vertelt aan uw partner. De onderzoeker praat met u over geschikte voorbehoedmiddelen.

Wordt u tijdens het onderzoek toch zwanger? Laat dit dan direct weten aan de onderzoeker.

6. Mogelijke bijwerkingen

Bij het botbiopt wordt er na verdoving van huid en onderliggende weefsels door de huid, een stukje uit het bot gehaald. De risico's bij dit onderzoek zijn gering. Er kan lokaal een bloeduitstorting ontstaan. Daarnaast is er een geringe kans op infectie.

Er kan mogelijk een allergische reacties op treden bij een MRI met gadolinium-contrast. De kans hierop is echter klein, namelijk 0,07%. Mocht behandeling van de allergische reactie nodig zijn, dan zal deze direct plaatsvinden.

De afname van de rectale swabs (zie punt 4) is pijnloos en levert geen risico voor de gezondheid op.

Stralingsbelasting

Bij de PET/CT-scan maken we gebruik van röntgenstraling en radioactieve stoffen. De extra stralingsbelasting in dit onderzoek is ongeveer 5 mSv. Ter vergelijking: de achtergrondstraling in Nederland is ongeveer 2,5 mSv per jaar.

Als u vaker meedoet aan wetenschappelijk onderzoek met stralingsbelasting, bespreekt u dan met de onderzoeker of deelname op dit moment verstandig is.

De straling die tijdens het onderzoek wordt gebruikt kan leiden tot schade aan uw gezondheid. Dit risico is echter klein. Wel adviseren we u de komende tijd niet nogmaals aan een wetenschappelijk onderzoek met stralingsbelasting mee te doen. Onderzoek of behandeling met straling om een medische reden is geen bezwaar.

7. Mogelijke voor- en nadelen

Het is belangrijk dat u de mogelijke voor- en nadelen goed afweegt voordat u besluit mee te doen. Er zijn geen belangrijke voordelen of nadelen aan het standaard gedeelte van het onderzoek. De onderzoeken worden ook gedaan als u niet mee zou doen aan de studie (met uitzondering van de rectale swab en de vragenlijsten).

Een voordeel van deelname aan het extra onderzoek met PET/CT, MRI en tweede botbiopt is dat 3 of 6 weken na start met antibioticum er extra onderzoek plaatsvindt (botbiopt, MRI-scan en PET/CT-scan). Deze onderzoeken dragen extra bij aan een weloverwogen besluit of verder behandeld moet worden met antibiotica of de behandeling definitief gestopt kan worden.

Nadelen van meedoen aan het extra onderzoek met PET/CT, MRI en tweede biopt kunnen zijn:

- Er kan een bloeduitstorting optreden ter plaatse van het botbiopt. En u bent tijd kwijt aan een extra ziekenhuisbezoek (PET/CT-scan).
- De MRI scan en PET/CT-scan vinden plaats in Amsterdam UMC, locatie AMC.

Al deze zaken zijn hiervoor onder punt 4, 5 en 6 beschreven.

8. Als u niet wilt meedoen of wilt stoppen met het onderzoek

U beslist zelf of u meedoet aan het onderzoek. Deelname is vrijwillig.

Als u niet wilt meedoen, wordt u op de gebruikelijke manier behandeld aan uw voetwond.

Als u wel meedoet, kunt u zich altijd bedenken en toch stoppen, ook tijdens het onderzoek. U wordt dan weer op de gebruikelijke manier behandeld voor de wond aan uw voet. U hoeft niet te zeggen waarom u stopt. Wel moet u dit direct melden aan de onderzoeker.

De gegevens die tot dat moment zijn verzameld, worden gebruikt voor het onderzoek.

Als er nieuwe informatie over het onderzoek is die belangrijk voor u is, laat de onderzoeker dit aan u weten. U wordt dan gevraagd of u blijft meedoen.

9. Einde van het onderzoek

Uw deelname aan het onderzoek stopt als

- alle bezoeken zoals beschreven onder punt 4 voorbij zijn
- u zelf kiest om te stoppen
- u zwanger wordt
- de onderzoeker of uw behandelend arts het beter voor u vindt om te stoppen

- Amsterdam UMC locatie VUmc, de overheid of de beoordelende medisch-ethische toetsingscommissie, besluit om het onderzoek te stoppen.

Het hele onderzoek is afgelopen als alle deelnemers klaar zijn met de behandeling en een jaar later een vervolgbezoek gehad hebben.

Na het verwerken van alle gegevens informeert de onderzoeker u over de belangrijkste uitkomsten van het onderzoek als u dit aangegeven hebt op het toestemmingsformulier.

Als u wilt weten in welke groep u zat, kunt u dit aangeven op het toestemmingsformulier. De onderzoeker zal het u dan na afronding van het onderzoek (als alle deelnemers klaar zijn en de gegevens verwerkt) laten weten.

10. Gebruik en bewaren van uw gegevens en lichaamsmateriaal

Voor dit onderzoek worden uw persoonsgegevens en lichaamsmateriaal verzameld, gebruikt en bewaard. Het gaat om gegevens zoals uw naam, adres, geboortedatum en om gegevens over uw gezondheid. Voor dit onderzoek is botweefsel en weefsel uit de wond nodig, het extra buisje bloed en indien u aan dit deel van de studie wil deelnemen, vier maal een rectale swab. Het verzamelen, gebruiken en bewaren van uw gegevens en uw lichaamsmateriaal is nodig om de vragen die in dit onderzoek worden gesteld te kunnen beantwoorden en de resultaten te kunnen publiceren. Wij vragen voor het gebruik van uw gegevens en lichaamsmateriaal uw toestemming. Ook vragen wij uw toestemming voor opslag van lichaamseigen materiaal in een biobank behorende tot deze studie en de afdeling interne geneeskunde.

Vertrouwelijkheid van uw gegevens en lichaamsmateriaal

Om uw privacy te beschermen krijgen uw gegevens en uw lichaamsmateriaal een code. Uw naam en andere gegevens die u direct kunnen identificeren worden daarbij weggelaten. Alleen met de sleutel van de code zijn gegevens tot u te herleiden. De sleutel van de code blijft veilig opgeborgen in Amsterdam UMC, locatie VUmc en is alleen beschikbaar voor de hoofdonderzoeker en de uitvoerend onderzoekers en worden dus alleen in Nederland gebruikt. De gegevens en lichaamsmateriaal die naar de biobank en andere deelnemende partijen zoals de laboratoria voor de kweken worden gestuurd bevatten alleen de code, maar niet uw naam of andere gegevens waarmee u kunt worden geïdentificeerd. Ook in rapporten en publicaties over het onderzoek zijn de gegevens niet tot u te herleiden, maar alleen geanonimiseerd beschikbaar.

Toegang tot uw gegevens voor controle

Sommige personen kunnen toegang krijgen tot al uw gegevens. Ook tot de gegevens zonder code. Dit is nodig om te kunnen controleren of het onderzoek goed en betrouwbaar is uitgevoerd. Personen die ter controle inzage krijgen in uw gegevens zijn: de commissie die de veiligheid van het onderzoek in de gaten houdt, een controleur die voor VUmc werkt, nationale toezichthoudende autoriteiten, bijvoorbeeld, de Inspectie Gezondheidszorg en Jeugd . Zij houden uw gegevens geheim. Wij vragen u voor deze inzage toestemming te geven.

Informatie over onverwachte bevindingen

Tijdens dit onderzoek kan er bij toeval iets gevonden worden dat niet van belang is voor het onderzoek maar wel voor U. Als dit belangrijk is voor uw gezondheid, dan zult u op de hoogte worden gesteld uw behandelend arts. U kunt dan met uw huisarts of specialist bespreken wat er gedaan moet worden. Ook hiervoor geeft u toestemming. Wanneer u dit niet wilt kunt u niet aan dit onderzoek meedoen.

Bewaartermijn gegevens en lichaamsmateriaal

Uw gegevens worden maximaal 15 jaar bewaard bij de opdrachtgever (Amsterdam UMC locatie VUmc). Indien u hiervoor toestemming geeft wordt uw lichaamsmateriaal ook maximaal 15 jaar bewaard.

Bewaren en gebruik van gegevens en lichaamsmateriaal voor ander onderzoek

Uw gegevens en lichaamsmateriaal worden niet gelijk vernietigd. Het lichaamsmateriaal wordt bewaard om daar in de loop van het onderzoek nog nieuwe bepalingen te kunnen doen. Uw afgestane lichaamsmateriaal kan na afloop van dit onderzoek ook nog van belang zijn voor ander wetenschappelijk onderzoek op het gebied van botinfectie bij patiënten met suikerziekte of ander onderzoek verricht binnen de interne geneeskunde. Daarvoor zullen uw gegevens 15 jaar worden bewaard. Indien u hiervoor toestemming geeft wordt uw lichaamsmateriaal 15 jaar bewaard in de biobank van het betreffende onderzoek en de afdeling interne geneeskunde. U kunt op het toestemmingsformulier aangeven of u hier wel of niet mee instemt. Indien u hier niet mee instemt, of bijvoorbeeld geen rectale swab wil afnemen, kunt u gewoon deelnemen aan de rest van het huidige onderzoek.

Het kan zijn dat dit of ander onderzoek, nieuwe vragen oplevert. Vragen die aanleiding geven om meer onderzoek te doen bij patiënten met diabetes en een (doorgemaakte)

botinfectie aan de voet. Daarom zouden wij graag de mogelijkheid behouden u in de toekomst nog eens te benaderen. Ook hiervoor vragen wij u apart om toestemming.

Wat is een biobank?

Voor een academisch ziekenhuis is het van groot belang dat lichaamsmateriaal, zoals ontlasting of weefsels, van grote groepen patiënten en gezonde mensen voor medisch-wetenschappelijk onderzoek wordt verzameld. Dit lichaamsmateriaal wordt samen met (medische) gegevens opgeslagen en voor lange tijd bewaard. Dit noemen we een biobank. Op het moment van verzamelen is niet altijd bekend voor welk specifiek onderzoek het lichaamsmateriaal zal worden gebruikt. In het algemeen gaat het om onderzoek naar oorzaken van ziekten, onderzoek dat kan leiden tot betere diagnose van ziekten, onderzoek waardoor ziekten beter kunnen worden voorspeld en onderzoek dat kan bijdragen aan de ontwikkeling van nieuwe behandelingen.

In het Amsterdam UMC locatie VUmc zijn alle biobanken waarin onderzoekers lichaamsmateriaal en gegevens verzamelen onderdeel van Biobank VUmc. Deze organisatie registreert en beheert het lichaamsmateriaal en bewaakt de kwaliteit ervan. De materialen worden zo opgeslagen dat het langdurig te bewaren is en voor toekomstig onderzoek kan worden gebruikt. Meer informatie over Biobank VUmc is te vinden op: https://www.vumc.nl/afdelingen/BiobankVUmc/

Deelname aan deze biobank levert voor uzelf geen voordeel op. Wel kunnen uitkomsten van toekomstig onderzoek uw zorg en die van andere mensen in de toekomst verbeteren.

U krijgt geen specifieke informatie over de onderzoeken waarvoor uw lichaamsmateriaal en uw medische gegevens in de toekomst worden gebruikt en u kunt niet bepalen voor welk specifiek onderzoek uw medische gegevens en het lichaamsmateriaal gebruikt worden. Dit is in een grootschalig biobankonderzoek helaas onmogelijk.

Intrekken toestemming

U kunt uw toestemming voor gebruik van uw persoonsgegevens altijd weer intrekken. Dit geldt voor dit onderzoek en ook voor het bewaren en het gebruik voor het toekomstige onderzoek. De onderzoeksgegevens die zijn verzameld tot het moment dat u uw toestemming intrekt worden nog wel gebruikt in het onderzoek. Uw lichaamsmateriaal wordt na intrekking van uw toestemming vernietigd. Als er al metingen met dat lichaamsmateriaal zijn gedaan, dan worden die gegevens nog wel gebruikt.

Ook kunt u uw toestemming gedeeltelijk intrekken voor de opslag in de biobank.

Meer informatie over uw rechten bij verwerking van gegevens

Voor algemene informatie over uw rechten bij verwerking van uw persoonsgegevens kunt u de website van de Autoriteit Persoonsgegevens raadplegen.

Bij vragen over uw rechten kunt u contact opnemen met de verantwoordelijke voor de verwerking van uw persoonsgegevens. Voor dit onderzoek is dat:

Amsterdam UMC locatie VU Medisch Centrum (VUmc). Zie bijlage A voor contactgegevens.

Bij vragen of klachten over de verwerking van uw persoonsgegevens raden we u aan eerst contact op te nemen met de onderzoek locatie. U kunt ook contact opnemen met de Functionaris voor de Gegevensbescherming van de instelling VU Medisch Centrum (VUmc). (zie bijlage A voor contactgegevens) of de Autoriteit Persoonsgegevens.

Registratie van het onderzoek

Informatie over dit onderzoek is ook opgenomen in een overzicht van medisch-wetenschappelijke onderzoeken namelijk www.toetsingonline.nl. Daarin zijn geen gegevens opgenomen die naar u herleidbaar zijn.

11. Verzekering voor proefpersonen

Voor iedereen die meedoet aan dit onderzoek is een verzekering afgesloten. De verzekering dekt schade door het onderzoek. Niet alle schade is gedekt. In **bijlage B** vindt u meer informatie over de verzekering en de uitzonderingen. Daar staat ook aan wie u schade kunt melden.

12. Informeren huisarts en/of behandelend specialist

Wij sturen uw huisarts en/of behandelend specialist altijd een brief om te laten weten dat u meedoet aan het onderzoek. Dit is voor uw eigen veiligheid. Als u dit niet goed vindt, kunt u niet meedoen aan dit onderzoek. Bij vragen kunnen we contact opnemen met uw (huis)arts, bijvoorbeeld over uw medische geschiedenis of over medicijngebruik.

13. Geen vergoeding voor meedoen

Deelname aan het onderzoek kost u niets. Wel betaalt u uw verplichte eigen bijdrage en eigen risico voor de behandeling, omdat dit onderdeel van de reguliere zorg is (met andere woorden: ook zonder dit onderzoek moet u behandeld worden). Wel krijgt u een vergoeding voor uw extra reiskosten en parkeerkosten.

14. Heeft u vragen?

Bij vragen kunt u contact opnemen met Merel Gramberg onderzoeker/promovenda VUmc
Voor onafhankelijk advies over meedoen aan dit onderzoek kunt u terecht bij een onafhankelijke arts. Hij weet veel over wetenschappelijk onderzoek en heeft ook verstand van de inhoud, maar hij is niet betrokken bij het uitvoeren van het onderzoek en hij heeft er ook geen belang bij.

Indien u klachten heeft over het onderzoek, kunt u dit bespreken met de onderzoeker of uw behandelend arts. Wilt u dit liever niet, dan kunt u zich wenden tot de klachtenfunctionaris/klachtencommissie van uw ziekenhuis. Bij klachten ten aanzien van de privacy en verwerking van uw persoonsgegevens kunt u zich richten tot Functionaris Gegevensbescherming. Alle gegevens vindt u in **bijlage A**: Contactgegevens.

15. Ondertekening toestemmingsformulier

Wanneer u voldoende bedenktijd heeft gehad, wordt u gevraagd te beslissen over deelname aan dit onderzoek. Indien u toestemming geeft, zullen wij u vragen deze op de bijbehorende toestemmingsverklaring schriftelijk te bevestigen. Door uw schriftelijke toestemming geeft u aan dat u de informatie heeft begrepen en instemt met deelname aan het onderzoek.

Zowel uzelf als de onderzoeker ontvangen een getekende versie van deze toestemmingsverklaring. Indien het door spoed niet mogelijk is de behandeling uit te stellen tot u bedenktijd heeft gehad, kunt u voorlopige toestemming geven. In dat geval wordt u bij een volgende gelegenheid gevraagd of u de eerder gegeven toestemming wilt bevestigen.
Dank voor uw aandacht.

16. Bijlagen bij deze informatie

A. Contactgegevens

B. Informatie over de verzekering

C. Schema onderzoekshandelingen / omschrijving onderzoekshandelingen

D Toestemmingsformulier(en)

Bijlage A: contactgegevens voor Amsterdam UMC, locatie VUmc

Onderzoekartsen:
Drs. MCTT Gramberg, promovenda/ arts in opleiding tot internist
Drs. RS Lagrand, promovenda / revalidatiearts
VU medisch centrum
Afdeling Interne Geneeskunde
Kamer ZH4A35
Postbus 7057
1007MB, Amsterdam
Telefoon: 0625716415
Email: m.gramberg@amsterdamumc.nl / r.lagrand@amsterdamumc.nl

Hoofdonderzoeker:
Dr. EJG Peters, internist-infectioloog-acute geneeskundige
VU medisch centrum
Afdeling Interne Geneeskunde, sectie infectieziekten
Kamer ZH4A46
Postbus 7057
1007MB, Amsterdam
telefoon: 06 25716081
Email: e.peters@amsterdamumc.nl

Onafhankelijk arts:
Dr. D van Raalte, internist-endocrinoloog
VU medisch centrum
Afdeling Interne Geneeskunde, sectie endocrinologie
Kamer ZH4A58
Postbus 7057
1007MB, Amsterdam
Telefoon: 020 4440534

Klachten:

Contactgegevens servicecentrum p&z

VUmc Servicecentrum p&z

Hal polikliniek, (PK 0 Hal 08) klachten@vumc.nl

Informatie en advies: ma t/m vrij van 8.00-12.00 en 13.00-16.30 uur op telefoonnummer (020) 444 0700/444 3555. Of per e-mail: zorgsupport@vumc.nl

Informatie over gegevensbescherming/privacy:

Functionaris Gegevensbescherming VUmc: privacy@vumc.nl

Bijlage B: informatie over de verzekering

Voor iedereen die meedoet aan dit onderzoek, heeft VUmc een verzekering afgesloten. De verzekering dekt schade door deelname aan het onderzoek. Dit geldt voor schade tijdens het onderzoek of binnen vier jaar na het einde van uw deelname aan het onderzoek. Schade moet u binnen die vier jaar aan de verzekeraar hebben gemeld.

De verzekering dekt niet alle schade. Onderaan deze tekst staat in het kort welke schade niet wordt gedekt.

Deze bepalingen staan in het Besluit verplichte verzekering bij medisch-wetenschappelijk onderzoek met mensen. Dit besluit staat op www.ccmo.nl, de website van de Centrale Commissie Mensgebonden Onderzoek (zie 'Bibliotheek' en dan 'Wet- en regelgeving').

Bij schade kunt u direct contact leggen met de verzekeraar [of schaderegelaar].

De verzekeraar van het onderzoek is:<br>Naam: Onderlinge Waarborgmaatschappij CentraMed B.A.Adres:<br>Postbus 7374, 2701 AJ Zoetermeer<br>Telefoonnummer: 070-3017070<br>E-mail: info@centramed.nl<br>Polisnummer: 624.529.204

De verzekering biedt een dekking van € 650.000,- per proefpersoon en € 5.000.000,- voor het hele onderzoek en € 7.500.000,- per jaar voor alle onderzoeken van dezelfde opdrachtgever.

De verzekering dekt de volgende schade **niet**:

- schade door een risico waarover u in de schriftelijke informatie bent ingelicht. Dit geldt niet als het risico zich ernstiger voordoet dan was voorzien of als het risico heel onwaarschijnlijk was;
- schade aan uw gezondheid die ook zou zijn ontstaan als u niet aan het onderzoek had meegedaan;
- schade door het niet (volledig) opvolgen van aanwijzingen of instructies;
- schade aan uw nakomelingen, als gevolg van een negatief effect van het onderzoek op u of uw nakomelingen;
- schade door een bestaande behandelmethode bij onderzoek naar bestaande behandelmethoden.

Bijlage C – Overzicht metingen

Indien u heeft besloten mee te doen aan het onderzoek dan ziet het tijdschema met alle metingen er als volgt uit:

1. Uw arts stelt een voetwond met infectie van het bot vast:

Onderzoeken: röntgenfoto, wondkweek, voetdrukmeting, botbiopt, bloedprikken, MRI en/of een PET/CT.

Vraagt of u wil meedoen aan deze wetenschappelijke studie:

U krijgt de proefpersonen informatie.

2. U besluit mee te doen:

U tekent de toestemmingsverklaring.

U krijgt vragenlijsten mee die u thuis in mag vullen.

3. Indien u hiervoor toestemming geeft neemt u voorafgaand aan start van antibioticum een rectale swab af, na staken van de antibiotica, na 6 en na 12 maanden

4. U start met antibioticum na afname van het botbiopt en diep weefsel biopt. Bij bekend worden van de kweek wordt het antibioticum eventueel aangepast op basis van de uitslag van de weefselkweek of het botbiopt.

5. U komt circa 1 keer per 2 weken voor controle op het diabetische voetenspreekuur. Daar krijgt u de reguliere behandeling inclusief temperatuurmeting van de voet en soms een bloedafname.

6. Indien u hier extra toestemming voor geeft: Er wordt geloot en afhankelijk daarvan krijgt u 3 of 6 weken na start van de behandeling een MRI-scan, PET/CT-scan en een nieuw botbiopt.

8. Na 6 weken behandeling levert u een rectale swab in.

9. 6 En 12 maanden na het stoppen van het antibioticum zullen er tijdens normale controles op de diabetische voetenpoli weer metingen gedaan worden voor de studie. Onder andere voetdrukmetingen. Ook zal u dan de laatste rectale swabs inleveren.

Figuur 1, schematisch overzicht van de studie

Start:

- Tekenen toestemmingsverklaring door de patiënt
- Lichamelijk onderzoek voeten inclusief slagaders, zenuwen, temperatuur en schoenen
- Invullen vragenlijsten
- Standaard laboratorium onderzoek
- Röntgenonderzoek van de voet
- MRI en/ of PET-scan
- Onderzoek naar bacteriën

↓

Randomisatie:

- Indelen naar behandeling op basis van het botbiopt of diepe weefselkweek
- Optioneel: indeling naar PET-scan en herhaald botbiopt na 3 of na 6 weken antibiotische behandeling

↓

- Switch naar gerichte therapie op basis van resultaat kweek
- Reguliere controle op de diabetische voetenpoli elke 1 – 3 weken

↓

Optioneel:

3 of 6 weken na de start van de behandeling:

- PET-scan en MRI
- percutaan botbiopt

Standaard:

- Totaal 6 weken behandeling met antibiotica.
- Na staken antibiotica: afname materiaal voor darmbacteriën en laboratorium onderzoek naar biomarkers

↓

6 maanden:

- Uitkomst beoordelen
- afname materiaal voor darmbacteriën

↓

12 maanden:

- Uitkomst beoordelen
- afname materiaal voor darmbacteriën

Bijlage D: toestemmingsformulier proefpersoon

- **Wat veroorzaakt botinfecties bij personen met diabetes en hoe lang moet de infectie worden behandeld? BeBoP-studie.**
- Ik heb de informatiebrief gelezen. Ook kon ik vragen stellen. Mijn vragen zijn voldoende beantwoord. Ik had genoeg tijd om te beslissen of ik meedoe. Als ik onvoldoende tijd had zal mij bij een volgende gelegenheid gevraagd worden de toestemming te bevestigen.
- Ik weet dat meedoen vrijwillig is. Ook weet ik dat ik op ieder moment kan beslissen om toch niet mee te doen of te stoppen met het onderzoek. Daarvoor hoef ik geen reden te geven.
- Ik geef toestemming voor het informeren van mijn huisarts/specialist(en) die mij behandelt dat ik meedoe aan dit onderzoek
- Ik geef toestemming voor het opvragen van informatie bij mijn huisarts/specialist(en) die mij behandelt over mijn medische voorgeschiedenis.
- Ik geef toestemming voor het verzamelen en gebruiken van mijn gegevens/bloedmonsters/ lichaamsmateriaal voor de beantwoording van de onderzoeksvraag in dit onderzoek. Ik geef toestemming voor het opslaan van deze data gedurende 15 jaar.
- Ik weet dat voor de controle van het onderzoek sommige mensen toegang tot al mijn gegevens kunnen krijgen. Die mensen staan vermeld in deze informatiebrief. Ik geef toestemming voor die inzage door deze personen.
- Ik weet dat er toevalsbevindingen kunnen worden gedaan. Ik weet dat ik hier van op de hoogte zal worden gesteld. Ook geef ik toestemming voor het informeren van mijn huisarts en/of behandelend specialist over onverwachte bevindingen die van belang (kunnen) zijn voor mijn gezondheid.
- Ik weet dat ik niet zwanger mag worden tijdens het onderzoek.
- Ik geef □ **wel**

  □ **geen**

  toestemming om mijn persoonsgegevens langer te bewaren en te gebruiken voor toekomstig onderzoek op het gebied van mijn aandoening en/of de onderzochte behandelwijze.
- Ik geef □ **wel**

  □ **geen**

  toestemming om mijn lichaamsmateriaal na dit onderzoek te bewaren in de BonE BiOPsy Biobank en biobank interne geneeskunde Amsterdam UMC locatie VUmc en om dit later nog voor ander/meer onderzoek te gebruiken, zoals in de informatiebrief staat.
- Ik geef □ **wel**

  □ **geen**

  toestemming om mij na dit onderzoek opnieuw te benaderen voor een vervolgonderzoek.

- Ik geef □ **wel**

  □ **geen**

  toestemming om na 3 of 6 weken (afhankelijk van loting) extra onderzoeken te ondergaan, te weten een tweede PET/CT-scan, MRI-scan en botbiopt.
- Ik geef □ **wel**

  □ **geen**

  toestemming voor een zelf-afname van een rectale swab.
- Ik wil □ **wel**

  □ **niet**

  geïnformeerd worden over welke behandeling ik heb gehad/in welke groep ik zat.
- Ik wil meedoen aan dit onderzoek.

Naam proefpersoon:

Handtekening: Datum : __ / __ / __

Ik verklaar dat ik deze proefpersoon volledig heb geïnformeerd over het genoemde onderzoek.

Als er tijdens het onderzoek informatie bekend wordt die de toestemming van de proefpersoon zou kunnen beïnvloeden, dan breng ik hem/haar daarvan tijdig op de hoogte.

Naam onderzoeker (of diens vertegenwoordiger):

Handtekening: Datum: __ / __ / __

----

indien van toepassing:

Aanvullende informatie is gegeven door:

Naam:

Functie:

Handtekening: Datum: __ / __ / __

----

* Doorhalen wat niet van toepassing is.

De proefpersoon krijgt een volledige informatiebrief mee, samen met een getekende versie van het toestemmingsformulier.
