# Supplementary material for: Using a BonE BiOPsy (BeBoP) to determine the causative agent in persons with diabetes and foot osteomyelitis: study protocol for a multicentre, randomised controlled trial
Source: Trials. 2021 Aug 3;22:517. doi: 10.1186/s13063-021-05472-6 (PMC8335883; doi:10.1186/s13063-021-05472-6)
Supplement: Supplementary file 2 — Additional file 2:. Appendix 2 [file 13063_2021_5472_MOESM2_ESM.pdf]

BIOBANKPROTOCOL

Aanvraag voor het opzetten van een collectie van lichaamsmaterialen (biobank)

BonE BiOPsy Biobank

Versie	1
Datum	23-7-2018

1	**ALGEMENE GEGEVENS**	
1.1	Eindverantwoordelijke voor biobank unit pathologie	**Prof. Dr. HWM Niessen, patholoog**
	Eindverantwoordelijke voor biobank unit medische microbiologie	**Drs. AE Budding, medisch microbioloog**
	Eindverantwoordelijke voor biobank unit klinische Chemie	**Dr. CE Teunissen, klinisch chemicus**
1.2	Organisatie en afdeling	**Pathologie**
	Organisatie en afdeling	**Medische microbiologie**
	Organisatie en afdeling	**Klinische chemie**
1.3	E-mailadres	**jwm.niessen@vumc.nl**
	E-mailadres	**d.budding@vumc.nl**
	E-mailadres	**c.teunissen@vumc.nl**
1.4	Contactpersoon en hoofdverantwoordelijke biobank	**Dr. EJG Peters**
1.5	Functie	**Internist infectioloog**
1.6	Telefoonnummer	**64425/0625716081**

1.7	E-mailadres	**e.peters@vumc.nl**
1.8	Verrichter/opdrachtgever	indien dit niet het VUmc is
1.9	Subsidiegever	**Diabetes fonds**

**2**	**BIOBANKGEGEVENS**	
2.1	Titel biobank	**BonE BiOPsy biobank**
2.2	Acroniem biobank	**BeBoP biobank**
2.3	Soort biobank	☒ Nader gebruik (overgebleven na afname in het kader van zorg)<br>☒ De Novo (afname in het kader van wetenschappelijk onderzoek)
2.4	Betreft het een reeds bestaande of een nog aan te leggen biobank?	☐ Reeds bestaande<br>☒ Nog aan te leggen
2.5	Is de Biobank gekoppeld aan een (niet) WMO-plichtige studie?	☐ Nee<br>☒ Ja, WMO-plichtige studie met METC-nummer: **2018.394**<br>☐ Ja, niet-WMO plichtige studie met METC-nummer:<br>*Zo ja, wat is de status van de studie*: lopend/afgerond/nog niet begonnen
2.6	Is er een wetenschapscommissie betrokken bij de Biobank?	☐ Ja<br>☒ Nee<br>Indien ja, wat is de samenstelling van de commissie?
2.7	CMG-nummer	**CMG: 2018-3035**

2.8	Onderzoek specialisme *(niet voor 'nader gebruik' biobanken, meerdere antwoorden mogelijk)*:	
	☐ Hartaandoeningen<br>☐ Congenitale, familiaire en genetische aandoeningen<br>☐ Bloed- en lymfestelselaandoeningen<br>☐ Oogaandoeningen<br>☐ Evenwichtsorgaan- en ooraandoeningen<br>☐ Ademhalingsstelsel-, thorax- en mediastinumaandoeningen<br>☐ Maagdarmstelselaandoeningen<br>☐ Huid- en onderhuidaandoeningen<br>☐ Voedings- en metabole stoornissen<br>☒ Infecties en parasitaire aandoeningen<br>☐ Neoplasmata, benigne, maligne en niet-gespecificeerd<br>☐ Voorplantingsstelsel- en borstaandoeningen	☐ Endocriene aandoeningen<br>☐ Letsel, intoxicaties<br>☐ Chirurgische en medische verrichtingen<br>☐ Bloedvataandoeningen<br>☐ Zwangerschap, perinatale periode, puerperium<br>☐ Sociale omstandigheden<br>☐ Immuunsysteemaandoeningen<br>☐ Lever- en galaandoeningen<br>☐ Zenuwstelselaandoeningen<br>☐ Psychische aandoeningen<br>☒ anders: **humaan fecaal microbioom**
2.9	Projectsamenvatting + doelstelling	**Bij de BeBoP trial zal een prospectief gerandomiseerd onderzoek verricht worden naar osteomyelitis bij patiënten met diabetische voetulcera. Gezocht wordt naar een optimale diagnostische methode en behandelduur. De uitkomsten van antibiotisch behandelen op basis van een kweek van een diep weefselbiopt zullen vergeleken worden met behandelen op basis van een kweek van een botbiopt. Een groep patiënten zal 3 weken behandeld worden versus een groep die 6 weken behandeld wordt. Beeldvorming en/of biomarkers inclusief moleculaire technieken zullen gebruikt worden op te bepalem wat de optimale antibiotische behandelduur is en of deze technieken een voorspellende waarde hebben ten aanzien van het ontwikkelen van een recidief na 6 en 12 maanden. De invloed van langdurig antibiotica op het fecale microbioom zal beoordeeld worden door enkele malen een rectale swabs af te laten nemen en deze te**

		analyseren met 16S sequencing en IS-Pro. Microbiologische data verkregen tijdens de studie zal met elkaar vergeleken worden.
2.10	Een korte beschrijving van het type en de hoeveelheid af te nemen materiaal	- Bij aanvraag van de studie zal één buis met spijtserum worden afgenomen.<br>- Tweemaal een botbiopt. Dit zal bestaan uit enkele spicula tot maximaal enkele milimeters van een botpijpje.<br>- Er zal maximaal viermaal een rectale swab gecollecteerd worden.
2.11	Een korte motivatie voor het type en de hoeveelheid af te nemen materiaal	- Eén buis met spijtserum zal verkregen worden om in een later stadium biomarkers te kunnen bepalen.<br>- Het eerste botbiopt zal gebruikt worden voor microbiologische kweek, moleculaire microbiologie en histopathologisch onderzoek ter diagnostiek naar osteomyelitis. Het tweede biopt zal verricht worden ter controle na antibiotische behandeling. De hoeveelheid zal een standaard hoeveelheid zijn noodzakelijk voor diagnostiek. Er zal geen extra materiaal worden afgenomen. Het materiaal dat gebruikt zal worden voor moleculaire diagnostiek zal tot deze analyse worden opgeslagen in de biobank. Tevens zal het materiaal in de toekomst gebruikt kunnen worden voor meer onderzoek naar<br>- De recatle swabs zullen gebruikt worden voor het analyseren van het fecale microbioom voor en na antibiotische behandeling. Dit zal per keer 1 swab betreffen. Gedurende het onderzoek zullen de recatle swabs worden opgeslagen in de biobank zodat deze aan het einde van de studie in één keer geanalyseerd kunnen worden. Tevens zal het materiaal in de toekomst gebruikt kunnen worden voor meer onderzoek naar het fecale microbioom bij deze patiëntengroep en in combinatie/ vergelijking met andere patiëntgroepen.
2.12	Biobankpopulatie	Volwassen proefpersonene die geïncludeerd worden in de studie met osteomyelitis van de diabetische voet.
2.13	Aantal te includeren patiënten<br>Aantal te includeren gezonde personen<br>Totaal	80<br>0<br>+/- 30 *per jaar.*
2.14	Van welke leeftijdscategorie zijn de donoren? (meerdere antwoorden mogelijk)	☐ jonger dan 12 jaar<br>☐ 12 jaar of ouder, maar jonger dan 16 jaar<br>☒ 16 jaar of ouder, **ga door naar vraag 2.16**
2.15	Motivatie minderjarige donoren	Waarom is het materiaal van minderjarige donoren nodig om de doelstelling te behalen?
2.16	Zijn de donoren wilsbekwaam?	☒ wilsbekwaam, **ga door naar vraag 2.18**<br>☐ wilsonbekwaam
2.17	Motivatie wilsonbekwame donoren	Waarom is het lichaamsmateriaal van wilsonbekwame donoren nodig voor het bereiken van het onderzoeksdoel?
2.18	Inclusie criteria	- diabetes mellitus type 1 of type 2<br>- wilsbekwaam en in staat om de gegeven onderzoeksinformatie te begrijpen, het protocol te volgen en informed consent te verlenen<br>1 van de volgende:<br>- geïnfecteerde wond tot op de fascie/ spier/ pees/ bot<br>- positieve probe to bone test<br>- een afwijkende röntgenfoto van de aangedane voet<br>- bezinking > of gelijk aan 70 mm/hr<br>- tekenen van osteomyelitis op MRI<br>- bacteriële groei bij botbiopt/ weefselkweek

2.19	Exclusie criteria	**- noodzaak tot chirurgisch bot debridement of amputatie<br>- ischemie aan de aangedane extremiteit die chirurgisch niet onder controle is te krijgen<br>- immuun gecompromitteerde staat<br>- zwangerschap of borstvoeding<br>- levensverwachting korter dan 1 jaar<br>- systemische antibiotica in de week voorafgaande aan inclusie**

3	**TYPE MATERIAAL EN HOEVEELHEID (MEERDERE KEUZES MOGELIJK)**<br>Welk en hoeveel materiaal wilt u opslaan in de biobank?		
3.1	**WEEFSEL**		
	**Gewenst materiaal voor de biobank**	**Totaal aantal samples**	**Hoeveelheid per sample (bv. ml., cm³, gram)**
	☒ Weefsel voor invriezen verkregen via biopsie	**160**	**1 – 5 mg**
	☐ Weefsel voor invriezen verkregen via resectie		
	☐ Weefsel voor paraffine inbedding verkregen via biopsie		
	☐ Weefsel voor paraffine inbedding verkregen via resectie		
3.2	**CELLEN**		
	**Gewenst materiaal voor de biobank**	**Totaal aantal samples**	**Hoeveelheid per sample (bv. ml., cm³, gram)**
	☐ Cellen geïsoleerd uit bloed		
	☐ Cellen geïsoleerd uit beenmerg		
	☐ Cellen geïsoleerd uit resectieweefsel (via dissociatie)		
	☐ "Snap-frozen cells" i.v.m. DNA/RNA		
	☐ anders, namelijk: ...		

3.3	**LICHAAMS(VLOEI)STOFFEN**			
	**Gewenst materiaal in de biobank**	**Totaal aantal samples**	**Hoeveelheid per sample (bv. ml., cm³, gram)**	**Wijze van verkrijgen** *(bv. bestaande lijn of extra prikactie, invasief of door de Donor zelf afgenomen)*
	☐ Bloed			
	☒ Serum	**1**	**3,5 ml**	**Bij de standaard zorg behoort de afname van bloed middels een venapunctie. Tijdens deze noodzakelijke prikactie zal een extra buis serum verkregen worden. Er is derhalve geen sprake van een extra prikactie.**
	☐ Plasma (anticoagulans EDTA)			
	☐ Plasma (anticoagulans Citraat)			
	☐ Plasma (anticoagulans Heparine)			
	☐ Liquor			
	☐ Urine			
	☒ Feces	**320**	**nihil**	**Zelfafname**
	☐ Peritoneaal dialysaat			
	☐ Speeksel			
	☐ Drainage vocht			plura, abdomen, anders

	☐ Vaginaal uitstrijkje			
	☐ Anaal uitstrijkje			
	☐ Haar			
	☐ Nagels			
3.4	**DNA/RNA**			
	**Gewenst materiaal in de biobank**		**Totaal aantal samples**	**Hoeveelheid per sample (bv. ml., cm³, gram)**
	☐ DNA/RNA geïsoleerd uit speeksel			
	☐ DNA/RNA geïsoleerd uit bloed			
	☐ DNA/RNA geïsoleerd uit beenmerg			
	☐ DNA/RNA geïsoleerd uit liquor			
	☒ DNA/RNA geïsoleerd uit weefsel<br>NB het betreft bacterieel DNA dat we uit het middels botbiopt verkregen materiaal willen isoleren. Het betreft dus geen patiënt specifiek humaan RNA/DNA.		**160**	**1-5 mg**

**4**	**BELASTING**<br>Waaruit bestaat de (tijds)belasting van deelname aan de biobank (en een eventueel daaraan voorafgaande screening) voor donoren?	
4.1	Tijdsbeslag: per bezoek	**Geen**
4.2	Aantal bezoeken	**Geen extra bezoeken**
4.3	Beschrijf in hoeverre donoren worden onderworpen aan handelingen dan wel een gedragswijze krijgen opgelegd, zoals vragenlijst, interviews, lichamelijk/psychologisch onderzoek, ontzegging, dieet.	**- Binnen de WMO plichtige studie wordt een tweede botbiopt afgenomen. Dit wordt niet extra verricht voor de biobank. Dit weefsel zal wel opgeslagen worden in de biobank.<br>- Binnen de WMO plichtige studie wordt een spijtbuis afgenomen ten tijden van een reguliere bloedafname. Dit wordt niet extra verricht voor de biobank. Dit weefsel zal wel opgeslagen worden in de biobank.<br>- Binnen de WMO plichtige studie worden enkele malen rectale swabs afgenomen ten tijden van een reguliere controles. Dit wordt niet extra verricht voor de biobank. Dit weefsel zal wel opgeslagen worden in de biobank.**
4.4	Worden de donoren getest op bepaalde aandoeningen/condities (bv. HIV, zwangerschap)?	☐ ja (motiveer), namelijk<br>☒ nee
4.5	Geef aan of en welke risico's er voor proefpersonen zijn verbonden aan deelname aan de biobank.	**Geen**
4.6	Waarom acht u de risico's van de afname minimaal en de belasting evenredig met het doel van het (toekomstige) onderzoek waarvoor juist dit type lichaamsmateriaal beschikbaar moet komen?	**N.v.t.**

**5**	**TOESTEMMING EN INTREKKING**<br>Beschrijf de werving en toestemmingsprocedure door onderstaande vragen te beantwoorden. De biobankinformatiebrief, toestemmingsformulier en intrekkingsformulier graag bijvoegen. Voor modeldocumenten zie de website van de TcB	
5.1	Werving	**Toestemming voor de BonE BiOPsy trial biobank wordt verkregen, samen met toestemming voor het protocol 'Determining the causative agent and optimal duration of antibiotic therapy in diabetic patients with foot osteomyelitis: BonE BiOPsy (BeBoP) trial' tijdens**

		**het bezoek aan het Voetwondenspreekuur voor reguliere zorg. Naast het toestemmingsformulier is ook een formulier voor het intrekken van eerder verleende toestemming beschikbaar. Dit wordt aan patiënten meegegeven. Lichaamsmaterialen zullen worden bewaard voor tenminste 15 jaar, of tot het materiaal niet meer bruikbaar is voor onderzoeksdoeleinden of de financiële middelen voor behoud van de biobank opdrogen. Patiënten hebben op ieder moment de mogelijkheid om van verdere deelname aan het onderzoek af te zien, waarbij al het in de biobank aanwezige lichaamsmateriaal dat reeds is verzameld zal worden vernietigd. De gegevens die al in een wetenschappelijk onderzoek zijn verwerkt kunnen niet worden vernietigd.**
5.2	Hoeveel bedenktijd krijgen de donoren/wettelijke vertegenwoordigers om te beslissen over deelname?	**Proefpersonen krijgen 24 uur de tijd om te beslissen of zij deel willen nemen aan de BeBoP trial. Deze duur zal ook gehandhaafd worden ten aanzien van opslag van lichaamseigen materiaal in de biobank.**
5.3	Kunnen donoren tijdens deelname aan de biobank opnieuw benaderd worden (bijvoorbeeld voor nader onderzoek of follow-up)?	☒ ja<br>(hier dient dan expliciet toestemming voor gevraagd te worden)<br>☐ nee
5.4	Zijn er criteria op basis waarvan de onderzoeker de deelname van de donor aan de biobank zou willen beëindigen (intrekking)	☐ ja, namelijk<br>☒ nee

**6**	**OPSLAG VAN BIOMATERIALEN EN GEGEVENS EN PRIVACY**<br>Procedures voor de codering en opslag van (persoons)gegevens en biomaterialen	
	**Lichaamsmateriaal**	
6.1	De Toetsingscommissie Biobank gaat er van uit dat het materiaal wordt aangemeld bij de Biobank Centrale van het VU medisch centrum. Zo niet, wat is hiervan de reden?	**n.v.t.**
6.2	Wordt het lichaamsmateriaal gecodeerd bewaard?	☒ ja<br>☐ nee, omdat
6.3	Wie draagt er zorg voor de codering/anonimisering?	☐ Biobank VUmc, **ga door met 6.7**<br>☒ de afdelingen verantwoordelijk voor de biobank<br>☐ anders, nl.:
6.4	Hoe is deze codering opgebouwd?	**- Op de afd. pathologie krijgt het materiaal een E-nummer toebedeeld.<br>- Op de afdeling medische microbiologie krijgt het materiaal een random nummerieke code toebedeeld door het programma glims.<br>- Op de afdeling klinische chemie krijgt het materiaal een random nummerieke code toebedeeld door het programma Item tracker.**
6.5	Wie heeft toegang tot de sleutel van deze code?	**- Biobank pathologie<br>- Alleen mensen die bevoegd zijn om in het programma glims te kijken.<br>- Biobank KCL**
6.6	Waar wordt de sleutel bewaard?	**- Afdeling pathologie<br>- Afdeling medische microbiologie<br>- Afdeling KCL**
6.7	Hoe lang wordt het lichaamsmateriaal maximaal bewaard?	**Maximaal 15 jaar**
6.8	Motivatie bewaartermijn	**Conform wettelijke bewaartermijn als beschreven voor medische gegevens (WGBO).**
	**Gegevens**	

6.9	Worden er (persoons)gegevens gekoppeld aan het materiaal?	☒ ja<br>☐ nee
6.10	Worden de gegevens gecodeerd bewaard?	☒ ja<br>☐ nee, omdat
6.11	Wie draagt er zorg voor de codering/anonimisering?	☐ Biobank VUmc, **ga door met 6.15**<br>☐ de afdeling verantwoordelijk voor de biobank<br>☒ anders, nl.: **de onderzoekers van de studie**
6.12	Hoe is deze codering opgebouwd?	**De patiënt krijgt een nummerieke studiecode.**
6.13	Wie heeft toegang tot de sleutel van deze code?	**Alleen de arts-onderzoekers**
6.14	Waar wordt de sleutel bewaard?	**Op de afdeling interne geneeskunde**
6.15	Hoe lang worden de gegevens maximaal bewaard?	**15 jaar**
6.16	Motivatie bewaartermijn	**Conform wettelijke bewaartermijn als beschreven voor medische gegevens (WGBO).**

**7**	**IDEEËN VOOR MOGELIJK ONDERZOEK**<br>Indien er ideeën zijn voor mogelijk onderzoek, of eventuele plannen wat betreft samenwerking met de industrie, graag kort benoemen.
	**- Verschil in fecaal microbioom na verchillende duur van antibiotische therapie.**<br>**- Verschil in fecaal microbioom na verschillende typen antibiotische therapie.**<br>**- Nieuwe biomarkers die indicatief zijn voor recidief en remissie van een osteomyelitis bij patiënten met diabetes.**

8	**ONDERTEKENING (eindverantwoordelijk onderzoeker)**	
8.1	Ondergetekende verklaart hierbij…<br>▪ …dit formulier naar waarheid te hebben ingevuld<br>▪ …kennis te hebben genomen van het Reglement Toetsing Biobank VUmc, en de daarin beschreven procedures, voorwaarden en richtlijnen t.a.v. de inleg, de uitgifte en het gebruik van lichaamsmaterialen en bijbehorende (klinische) gegevens	
8.2	Naam:	**EJG Peters**
	Functie:	**MD, PhD, infectioloog**
	Afdeling:	**Interne geneeskunde, sectie infectieziekten**
	Datum:	
	Handtekening:<br><br>Indien er handtekeningen ontbreken dan wordt het formulier niet in behandeling genomen.	

9	**ONDERTEKENING (eindverantwoordelijk afdelingshoofd voor de biobank)**	
9.1	Ondergetekende verklaart hierbij namens biobank:	**BonE BiOPsy trial Biobank**

	▪ ...akkoord te gaan met het opslaan van bovengenoemd lichaamsmateriaal voor bovengenoemd doel<br>▪ ... verantwoordelijk te zijn voor bovengenoemde biobank<br>▪ ...dat bovengenoemd onderzoek past binnen de doelen van het wetenschapsbeleid van de divisie en VUmc	
9.2	Naam:	**JWM Niessen/ D Budding/ CE Teunissen**
	Afdeling:	**Pathologie/ medische microbiologie/ klinische chemie**
	Datum:	
	Handtekening:	30/7/18 26-7-2018
	**Indien er handtekeningen ontbreken dan wordt het formulier niet in behandeling genomen.**	

Bijgevoegd vindt u de documenten behorend bij de indiening.

Bijlagen	Bijgevoegd	N.v.t.
C1 Biobankprotocol *	x	
E1 Informatiebrief *	x	
E2 Toestemmingsverklaring *	x	
E2 Intrekkingsformulier *	x	
Advies Wetenschapscommissie		x
**Overige bijlagen**	**Bijgevoegd**	

* Verplichte documenten
